# Adenovirus DNA is detected at increased frequency in Guthrie cards from children who develop acute lymphoblastic leukaemia

**DOI:** 10.1038/sj.bjc.6603983

**Published:** 2007-09-18

**Authors:** B Gustafsson, W Huang, G Bogdanovic, F Gauffin, A Nordgren, G Talekar, D A Ornelles, L R Gooding

**Affiliations:** 1 Department of Pediatrics, Karolinska University Hospital, Karolinska Institutet, Stockholm, Sweden; 2 Department of Microbiology and Immunology, Emory University School of Medicine, Atlanta, GA, USA; 3 Department of Clinical Microbiology, Karolinska University Hospital, Karolinska Institutet, Stockholm, Sweden; 4 Department of Clinical Genetics, Karolinska University Hospital, Karolinska Institutet, Stockholm, Sweden; 5 Department of Microbiology and Immunology, Wake Forest University School of Medicine, Winston-Salem, NC, USA

**Keywords:** ALL, adenovirus, human, prenatal infection, Guthrie cards

## Abstract

Epidemiological evidence suggests that childhood acute lymphoblastic leukaemia (ALL) may be initiated by an in infection *in utero*. Adenovirus DNA was detected in 13 of 49 neonatal blood spots from ALL patients but only in 3 of 47 controls (*P*=0.012) suggesting a correlation between prenatal adenovirus infection and the development of ALL

Acute lymphoblastic leukaemia (ALL) is the most common malignancy in children (Greenlee *et al*, 2000). Recent studies have shed light on the natural history of this disease. Identification of leukaemia-associated translocations in neonatal blood spots (Guthrie cards) of children who will develop leukaemia years later indicates that the initiating event(s) of ALL usually occur before birth (Gale *et al*, 1997; Wiemels *et al*, 1999). Epidemiologic studies (Kinlen, 1988; Kinlen and Doll, 2004) suggest that at least one step in the process of ALL development involves an infectious agent (reviewed in Greaves, 2006). The hallmark genetic mutations, notably translocations, of childhood ALL arise when repair of DNA breaks is faulty or inhibited. Infection with some DNA viruses, notably herpesviruses and adenoviruses, interferes with cellular DNA repair mechanisms (Weitzman and Ornelles, 2005; Wilkinson and Weller, 2006) and could lead to these characteristic genetic changes. To determine whether a prenatal virus infection could lead to the development of pre-leukaemic cells containing characteristic translocations, a correlation is sought between detection of viral DNA in Guthrie cards and the eventual development of leukaemia. This approach showed no association between infection with polyoma viruses (Priftakis *et al*, 2003), herpesviruses (Bogdanovic *et al*, 2004; Gustafsson *et al*, 2006), or parvovirus B19 (Isa *et al*, 2004) and childhood ALL.

The common, endemic species C adenoviruses (serotypes 1, 2, 5, and 6) normally cause respiratory tract infections in young children (Brandt *et al*, 1969; Avila *et al*, 1989) and persistent infection of lymphoid tissues (Garnett *et al*, 2002). We investigated whether adenovirus infections could be correlated with ALL by studying Guthrie cards from children who later developed ALL and healthy controls.

## MATERIALS AND METHODS

### Guthrie cards

Neonatal capillary blood spots from newborn Swedish infants were used for this study. These blood spots were collected at 3–5 days of age. Four droplets of capillary blood were taken and blotted onto filter paper (Guthrie card). One blood spot contains about 3 × 10^4^ nucleated cells. Guthrie cards were stored at 4°C.

### Patients

Guthrie cards were identified from 49 children born between 1977 and 1998, who were diagnosed with ALL between 1980 and 2001 (Table 2). As a control group Guthrie cards from 47 healthy children, matched for birth date and birthplace, were analysed, using the Swedish Medical Birth Register to identify these children. Written informed consent was obtained from all patients, controls and parents. The local ethics committee of the Karolinska Institutet at the Karolinska University Hospital approved the study protocol.

### DNA extraction from Guthrie cards

Three uniform discs, 3 mm in diameter, were punched from one of the four blood spots from the stored material and the DNA extracted as previously described with minor modifications (Barbi *et al*, 1996).

### PCR assays

Two different nested PCR assays were used in this study (nucleotides 18 838–19 205 and 20 721–21 572 of the Ad2 sequence (RefSeq AC000007)). Both assays amplified DNA from all species C serotypes, but not from members of other adenovirus species (A, B, D, E, and F). In some cases, samples were also tested with a quantitative real-time PCR assay (Garnett *et al*, 2002). As a control, PCR with a set of HLA-DQ alpha primers was performed as described (Saiki *et al*, 1986).

### **Statistical analysis**

Categorical parameters were evaluated using the Fisher exact test and the Cochran–Mantel–Haenszel test. Logistic regression was used to determine if the clinical outcomes were jointly associated with patient characteristics and adenovirus DNA.

## RESULTS

DNA extracted from Guthrie cards from 49 ALL patients and from 47 normal controls was analysed for the presence of species C adenovirus DNA. Individual samples were assayed on at least three separate occasions using two different PCR assays. Thirteen of the ALL samples and three of the control samples contained amplifiable adenovirus DNA (Table 1). These results show a significant association (*P*=0.012) between species C adenovirus DNA and leukaemia. The odds ratio shows a 5.2-fold (95% CI:1.3–31) greater likelihood of detecting species C adenovirus DNA in the neonatal blood of a child that subsequently develops ALL than in the neonatal blood of a child that does not develop ALL.

Adenovirus DNA levels in positive patient samples were quantified by real-time PCR, which is slightly less sensitive than the nested assays. Five of the 13 ALL samples and none of the three adenovirus DNA-positive control samples contained detectable DNA by real-time PCR (data not shown). The range of DNA copies in these assays was from 1 to 30 with a median of 2.3 copies.

Contingency tables and logistic regression were used to determine if the detection of adenovirus DNA was associated with any patient characteristics or clinical outcomes summarised in Table 2. A non-random (*P*=0.032) distribution of adenovirus DNA among risk groups was observed. However, the very limited number of adenovirus-positive samples analysed here requires further investigation to determine if this distribution is indeed non-random and if the exclusion of adenovirus DNA from the Guthrie cards of patients in the intermediate-risk group is significant. No other relationship (*P*>0.5) was found between adenovirus DNA in the neonatal blood spot and the remaining characteristics identified in Table 2.

## DISCUSSION

For species C adenovirus to contribute to the earliest steps in development of childhood leukaemia, this virus must infect the fetus at least as frequently as the incidence of neonatal pre-leukaemic clones in the population, estimated at 1–5% (Mori *et al*, 2002). In addition to being a likely fetal pathogen (Towbin *et al*, 1994; Van den Veyver *et al*, 1998; Oyer *et al*, 2000; Baschat *et al*, 2003; Reddy *et al*, 2005), adenovirus DNA is also detected in the amniotic fluid from apparently normal pregnancies. Of 1187 unique samples in four large studies, 64 (5.4%) contained adenoviral DNA (Van den Veyver *et al*, 1998; Wenstrom *et al*, 1998; Baschat *et al*, 2003; Reddy *et al*, 2005), which compares favourably with 6% adenovirus DNA-positive samples among the normal controls in the present study (Table 1). And, as is the case with children infected post-natally, adenoviral DNA is retained in latently infected T lymphocytes for years following primary infection (Garnett *et al*, 2002). Thus, the finding of species C adenovirus in neonatal blood spots is evidence of prenatal infection, as is the case for other prenatal viral infections (Barbi *et al*, 1996; Johansson *et al*, 1997; Fischler *et al*, 1999).

Adenovirus DNA sequences have not been detected in cell lines derived from childhood ALL (LR Gooding, unpublished), and, indeed, MacKenzie *et al* (2006) recently reported that no potential ‘exogenous’ DNA could be detected in leukaemia cells by representational difference analysis, indicating that no DNA virus was present in the cells. Gene products of adenovirus can transform cells in culture by a hit-and-run mechanism that leaves no viral DNA in the transformed cells (Nevels *et al*, 2001). Because species C adenoviruses profoundly block double-stranded DNA-break repair (Weitzman and Ornelles, 2005), it is conceivable that infection with species C adenovirus could lead to the acquisition of genomic mutations common to childhood ALL.

The finding of a significantly elevated frequency of species C adenovirus DNA in Guthrie cards of children who later developed ALL suggests a potential role for adenovirus at an early stage in the development of this disease. Further research is necessary to determine if this species has a causative role in this disease.

## Figures and Tables

**Table 1:** Adenovirus DNA detected in Guthrie cards of ALL patients and controls

	**Adenovirus DNA status**		
**Disease status**	**Positive**	**Negative**	**% Adenovirus DNA-positive**
ALL	13	36	27
Normal	3	44	6

ALL=acute lymphoblastic leukaemia.

**Table 2:** Patient characteristics

**Characteristic**	**No.**	**Adenovirus DNA**
*Diagnosis*		
Pre-B ALL	44	11
B ALL	1	0
T ALL	4	2
		
*Sex*		
Female	25	6
Male	24	7
		
*Risk group*		
Standard risk	20	8
Intermediate risk	12	0
High risk	17	5
		
*Karyotype*		
Hyperdiploid	23	7
t(9;22)(q34;q11)	3	1
t(10;11)(?;q23)	1	0
t(12;21)(p12;q21)	1	0
t(1;19)(q23;p13)	1	0
t(Y;15)(p11.23;q11.2)	1	1
inv(17q)	1	1
add(1), add(7)	1	0
add(6)(q27)	1	0
No abnormality detected	10	3
Inconclusive	17	6
		
*Outcome*		
Disease-free >5 years	32	10
Stem cell transplantation	23	8
Deceased	7	2

ALL=acute lymphoblastic leukaemia.

## References

[bib1] Avila MM, Carballal G, Rovaletti H, Ebekian B, Cusminsky M, Weissenbacher M (1989) Viral etiology in acute lower respiratory infections in children from a closed community. Am Rev Respir Dis 140: 634–637 2675703 10.1164/ajrccm/140.3.634

[bib2] Barbi M, Binda S, Primache V, Luraschi C, Corbetta C (1996) Diagnosis of congenital cytomegalovirus infection by detection of viral DNA in dried blood spots. Clin Diagn Virol 6: 27–32 15566887 10.1016/0928-0197(96)00228-0

[bib3] Baschat AA, Towbin J, Bowles NE, Harman CR, Weiner CP (2003) Prevalence of viral DNA in amniotic fluid of low-risk pregnancies in the second trimester. J Matern Fetal Neonatal Med 13: 381–384 12962262 10.1080/jmf.13.6.381.384

[bib4] Bogdanovic G, Jernberg AG, Priftakis P, Grillner L, Gustafsson B (2004) Human herpes virus 6 or Epstein–Barr virus were not detected in Guthrie cards from children who later developed leukaemia. Br J Cancer 91: 913–915 15292925 10.1038/sj.bjc.6602099 PMC2409878

[bib5] Brandt C, Kim H, Vargosko A, Jeffries B, Arrobio J, Rindge B, Parrott R, Chanock R (1969) Infections in 18 000 infants and children in a controlled study of respiratory tract disease. I. Adenovirus pathogenicity in relation to serologic type and illness syndrome. Am J Epidemiol 90: 484–500 4312064 10.1093/oxfordjournals.aje.a121094

[bib6] Fischler B, Rodensjo P, Nemeth A, Forsgren M, Lewensohn-Fuchs I (1999) Cytomegalovirus DNA detection on Guthrie cards in patients with neonatal cholestasis. Arch Dis Child Fetal Neonatal Ed 80: F130–F134 10325791 10.1136/fn.80.2.f130 PMC1720908

[bib7] Gale KB, Ford AM, Repp R, Borkhardt A, Keller C, Eden OB, Greaves MF (1997) Backtracking leukemia to birth: identification of clonotypic gene fusion sequences in neonatal blood spots. Proc Natl Acad Sci USA 94: 13950–13954 9391133 10.1073/pnas.94.25.13950 PMC28413

[bib8] Garnett CT, Erdman D, Xu W, Gooding LR (2002) Prevalence and quantitation of species C adenovirus DNA in human mucosal lymphocytes. J Virol 76: 10608–10616 12368303 10.1128/JVI.76.21.10608-10616.2002 PMC136639

[bib9] Greaves M (2006) Infection, immune responses and the aetiology of childhood leukaemia. Nat Rev Cancer 6: 193–203 16467884 10.1038/nrc1816

[bib10] Greenlee RT, Murray T, Bolden S, Wingo PA (2000) Cancer statistics, 2000. CA Cancer J Clin 50: 7–33 10735013 10.3322/canjclin.50.1.7

[bib11] Gustafsson B, Jernberg AG, Priftakis P, Bogdanovic G (2006) No CMV DNA in Guthrie cards from children who later developed ALL. Pediatr Hematol Oncol 23: 199–205 16517536 10.1080/08880010500506677

[bib12] Isa A, Priftakis P, Broliden K, Gustafsson B (2004) Human parvovirus B19 DNA is not detected in Guthrie cards from children who have developed acute lymphoblastic leukemia. Pediatr Blood Cancer 42: 357–360 14966833 10.1002/pbc.20001

[bib13] Johansson PJ, Jonsson M, Ahlfors K, Ivarsson SA, Svanberg L, Guthenberg C (1997) Retrospective diagnostics of congenital cytomegalovirus infection performed by polymerase chain reaction in blood stored on filter paper. Scand J Infect Dis 29: 465–468 9435033 10.3109/00365549709011855

[bib14] Kinlen L (1988) Evidence for an infective cause of childhood leukaemia: comparison of a Scottish new town with nuclear reprocessing sites in Britain. Lancet 2: 1323–1327 2904050 10.1016/s0140-6736(88)90867-7

[bib15] Kinlen L, Doll R (2004) Population mixing and childhood leukaemia: fallon and other US clusters. Br J Cancer 91: 1–3 15226760 10.1038/sj.bjc.6601982 PMC2364755

[bib16] MacKenzie J, Greaves MF, Eden TO, Clayton RA, Perry J, Wilson KS, Jarrett R.F (2006) The putative role of transforming viruses in childhood acute lymphoblastic leukemia. Haematologica 91: 240–243 16461310

[bib17] Mori H, Colman SM, Xiao Z, Ford AM, Healy LE, Donaldson C, Hows JM, Navarrete C, Greaves M (2002) Chromosome translocations and covert leukemic clones are generated during normal fetal development. Proc Natl Acad Sci USA 99: 8242–8247 12048236 10.1073/pnas.112218799 PMC123052

[bib18] Nevels M, Tauber B, Spruss T, Wolf H, Dobner T (2001) ‘Hit-and-run’ transformation by adenovirus oncogenes. J Virol 75: 3089–3094 11238835 10.1128/JVI.75.7.3089-3094.2001 PMC114102

[bib19] Oyer CE, Ongcapin EH, Ni J, Bowles NE, Towbin JA (2000) Fatal intrauterine adenoviral endomyocarditis with aortic and pulmonary valve stenosis: diagnosis by polymerase chain reaction. Hum Pathol 31: 1433–1435 11112222

[bib20] Priftakis P, Dalianis T, Carstensen J, Samuelsson U, Lewensohn-Fuchs I, Bogdanovic G, Winiarski J, Gustafsson B (2003) Human polyomavirus DNA is not detected in Guthrie cards (dried blood spots) from children who developed acute lymphoblastic leukemia. Med Pediatr Oncol 40: 219–223 12555248 10.1002/mpo.10246

[bib21] Reddy UM, Baschat AA, Zlatnik MG, Towbin JA, Harman CR, Weiner CP (2005) Detection of viral deoxyribonucleic acid in amniotic fluid: association with fetal malformation and pregnancy abnormalities. Fetal Diagn Ther 20: 203–207 15824499 10.1159/000083906

[bib22] Saiki RK, Bugawan TL, Horn GT, Mullis KB, Erlich HA (1986) Analysis of enzymatically amplified beta-globin and HLA-DQ alpha DNA with allele-specific oligonucleotide probes. Nature 324: 163–166 3785382 10.1038/324163a0

[bib23] Towbin JA, Griffin LD, Martin AB, Nelson S, Siu B, Ayres NA, Demmler G, Moise KJ, Zhang YH (1994) Intrauterine adenoviral myocarditis presenting as nonimmune hydrops fetalis: diagnosis by polymerase chain reaction. Pediatr Infect Dis J 13: 144–150 8190541 10.1097/00006454-199402000-00013

[bib24] Van den Veyver IB, Ni J, Bowles N, Carpenter Jr RJ, Weiner CP, Yankowitz J, Moise Jr KJ, Henderson J, Towbin JA (1998) Detection of intrauterine viral infection using the polymerase chain reaction. Mol Genet Metab 63: 85–95 9562961 10.1006/mgme.1997.2651

[bib25] Weitzman MD, Ornelles DA (2005) Inactivating intracellular antiviral responses during adenovirus infection. Oncogene 24: 7686–7696 16299529 10.1038/sj.onc.1209063

[bib26] Wenstrom KD, Andrews WW, Bowles NE, Towbin JA, Hauth JC, Goldenberg RL (1998) Intrauterine viral infection at the time of second trimester genetic amniocentesis. Obstet Gynecol 92: 420–424 9721782 10.1016/s0029-7844(98)00210-5

[bib27] Wiemels JL, Cazzaniga G, Daniotti M, Eden OB, Addison GM, Masera G, Saha V, Biondi A, Greaves MF (1999) Prenatal origin of acute lymphoblastic leukaemia in children. Lancet 354: 1499–1503 10551495 10.1016/s0140-6736(99)09403-9

[bib28] Wilkinson DE, Weller SK (2006) Herpes simplex virus type I disrupts the ATR-dependent DNA-damage response during lytic infection. J Cell Sci 119: 2695–2703 16757521 10.1242/jcs.02981 PMC4427570

